# Paraganglioma and phaeochromocytoma: a review of current guidelines and new diagnostic and therapeutic approaches

**DOI:** 10.1530/ERC-26-0310

**Published:** 2026-10-13

**Authors:** Ruth T Casey, Charlotte Lussey-Lepoutre

**Affiliations:** ^1^ Department of Genomic Medicine, University of Cambridge, Cambridge Biomedical Campus, Cambridge, UK; ^2^ Department of Endocrinology, Cambridge University Hospital, Cambridge Biomedical Research Centre, Addenbrooke’s Hospital, Cambridge, UK; ^3^ Sorbonne Université, Department of Nuclear Medicine, Hôpital Pitié Salpêtrière, APHP, Paris, France; ^4^ Sorbonne Université, Université Paris Cité, Inserm, Centre de Recherche des Cordeliers, Paris, France

**Keywords:** paraganglioma, *SDHB*, *SDHD*, guidelines

## Abstract

Paragangliomas and phaeochromocytomas, recently reclassified to unify the disease as extra-adrenal and intra-adrenal paragangliomas, are rare neuroendocrine tumours. These tumours have an exceptionally strong hereditary component, with up to 40% of cases being driven by germline mutations in susceptibility genes; therefore, a cornerstone of modern PPGL care is the genotype-first approach, which heavily dictates initial assessment, surgical strategy, and follow-up. In recent years, the clinical landscape has evolved rapidly, leading to the publication of more than ten new consensus statements since 2022. This review article highlights some of these major paradigm shifts including i) a transition towards genotype-tailored clinical management, ii) specialized paediatric diagnostic and management protocols, iii) updated histopathological criteria, iv) the use of advanced targeted therapeutics, and v) the integration of novel predictive tools.

## Introduction

Paragangliomas (PGLs) are rare neuroendocrine tumours arising from the paraganglia, which are composed of cells derived from the neural crest and are distributed throughout the autonomic nervous system (1). A phaeochromocytoma (PCC) originates specifically from the adrenal medulla. While they were recently renamed as intra-adrenal PGL, the term pheochromocytoma/PCC is retained in this review for readability. Paragangliomas can develop at multiple anatomical sites, including the head and neck region, where they are typically non-secretory or secrete dopamine exclusively and arise from parasympathetic paraganglia, as well as the thorax, abdomen, and pelvis, where they more frequently originate from sympathetic paraganglia and may produce catecholamines, such as noradrenaline, adrenaline, or dopamine (1).

The overall incidence of phaeochromocytomas and paragangliomas (PPGLs) is estimated at approximately 1–2 cases per 100,000 individuals per year, though this figure is likely underestimated given the increasing prevalence of incidentally discovered or genetically driven tumours. Indeed, one of the most distinctive features of PPGLs is their strong hereditary component: up to 40% of cases (including cases without syndromic presentations) are attributable to germline mutations in one of more than 20 susceptibility genes, including *SDHB*, *SDHD*, *SDHC*, *VHL*, *RET*, *NF1*, and *TMEM127*, among others. Another 30–40% of apparently sporadic tumours also carry somatic driver mutations (e.g. *VHL, RET, HRAS, EPAS1/HIF2A, NF1*), such that up to 70% of PPGL cases can be attributed to specific driver genetic alterations (2). This genetic heterogeneity has led to the reclassification of PGLs into distinct molecular clusters (Table 1) with specific clinical, biochemical, and prognostic implications (3, 4). Genetically determined PPGLs are regrouped into 2 of the 3 clusters (Table 1). Cluster 1, known as the ‘pseudo-hypoxic’ cluster, is divided into two sub-clusters. Cluster 1A comprises the syndromic von Hippel–Lindau disease (*VHL*) and *EPAS1* mutations. Cluster 1B mainly comprises genes involved in the TCA cycle (*FH, MDH2, DLST, GOT2, SLC25A11,* and *MDH2*); the *SDHx* genes encoding the succinate dehydrogenase enzyme (*SDHA, SDHB, SDHC, SDHD,* and *SDHAF2*) are responsible for about half of hereditary PGLs. Cluster 2 is a kinase-signalling cluster, comprising genes that predispose to PCC, frequently bilateral, in either a syndromic (*RET* in multiple endocrine neoplasia type 2 and *NF1* for type 1 neurofibromatosis, also known as von Recklinghausen’s disease) or nonsyndromic (*MAX, TMEM127*) presentation.

**Table 1:** Molecular classification of PPGLs.

Molecular cluster	Principal genes	Biochemical/molecular phenotype	Metastatic risk
Cluster 1 – Pseudohypoxia	TCA-cycle subgroup: *SDHA, SDHB, SDHC, SDHD, SDHAF2, FH, MDH2, DLST, GOT2, SLC25A11*; hypoxia-signalling subgroup: *VHL, EGLN1, EGLN2, EPAS1 (HIF2A)*	Stabilization of HIF signalling, facilitating pseudohypoxic transcriptional dysregulation, increased angiogenesis and metabolic reprogramming. Typically associated with a noradrenergic phenotype, tumours may also show increased 3-methoxytyramine	Moderate–high overall. Particularly high in *SDHB*-associated PPGLs; by contrast, *VHL*-dominant cluster 1B tumours generally have a lower metastatic risk
Cluster 2 – Kinase signalling	*RET, NF1, TMEM127, MAX, HRAS*; additional reported alterations include *MET, MERTK, FGFR1,* and *BRAF*	Activation of RAS/RAF/MEK/ERK, PI3K/AKT/mTOR, and related receptor-kinase pathways, promoting cell proliferation, survival, and growth. Associated with a noradrenergic and/or adrenergic biochemical phenotype, depending on the driver	Generally lower than cluster 1 and Wnt-altered disease. These tumours are often relatively well differentiated and have comparatively low metastatic potential
Cluster 3 – Wnt-altered	Somatic *CSDE1* mutations and *MAML3* fusions (including UBTF–MAML3); *ATRX* and *TERT* alterations may occur as additional aggressive features	Dysregulation of Wnt-related transcriptional programmes, with altered cellular differentiation, proliferation, and tumour-cell plasticity. *MAML3* fusion-positive tumours represent a distinctive molecular subgroup of sporadic PPGLs	High/relatively aggressive. The *MAML3* fusion in particular has been associated with metastatic PPGLs and is considered a molecular correlate of aggressive disease

Since the publication of the 2014 Endocrine Society clinical practice guideline, at least 10 society-sponsored or international consensus guidelines/statements addressing PPGLs have been published (Table 2), including guidance on gene-specific management (*SDHx*) (5, 6, 7) and management of PPGLs in children (8, 9) (Table 2). Additional expert clinical practice recommendations and guidelines have been published on the management of metastatic PPGLs (10, 11), head and neck paragangliomas (12), long-term follow-up (13), and histopathological reporting (14). In this review, we will focus on a selection of these key guidelines/consensus statements and clinical practice articles and their influence on clinical management. Finally, we will review some recent advances in clinical research studies, which may feature in future clinical guidelines and consensus statements as further data are curated (Fig. 1).

**Table 2:** Society-endorsed guidelines and international consensus statements on PPGLs published since 2014.

Year	Guidance	Society/group	Scope
2014	Pheochromocytoma and paraganglioma: An Endocrine Society clinical practice guideline	Endocrine Society	Broad PPGL diagnosis, treatment, and follow-up (13)
2019	European Association of Nuclear Medicine Practice Guideline/Society of Nuclear Medicine and Molecular Imaging procedure standard 2019 for radionuclide imaging of phaeochromocytoma and paraganglioma	EANM + SNMMI	Nuclear/functional imaging (23)
2020	Genetics, diagnosis, management and future directions of research of phaeochromocytoma and paraganglioma: a position statement and consensus of the Working Group on endocrine hypertension of the European Society of Hypertension	European Society of Hypertension	Broad PPGL genetics, diagnosis, management, and surveillance (1)
2021	International consensus on initial screening and follow-up of asymptomatic SDHx mutation carriers	International PPGL expert consensus	SDHA/B/C/D carriers; screening and surveillance (5)
2021	The North American Neuroendocrine Tumor Society Consensus Guidelines for surveillance and management of metastatic and/or unresectable phaeochromocytoma and paraganglioma	NANETS	Metastatic/unresectable PPGLs and surveillance (45)
2021	Multidisciplinary practice guidelines for the diagnosis, genetic counselling and treatment of pheochromocytomas and paragangliomas	Multiple Spanish societies (SEEN, SEOM, SERAM, SEMNIM, SEORL, SEAP, SEOR, AEC)	Broad diagnosis, genetics, and treatment (58)
2023/24	Clinical consensus guideline on the management of phaeochromocytoma and paraganglioma in patients harbouring germline SDHD pathogenic variants	International expert consensus	SDHD-associated PPGLs (7)
2023/24	Management of phaeochromocytoma and paraganglioma in patients with germline SDHB pathogenic variants: an international expert consensus statement	International PPGL expert consensus	SDHB-associated PPGLs (6)
2024	International consensus statement on the diagnosis and management of PPGL in children and adolescents	International multidisciplinary consensus	Paediatric PPGLs (8)
2025	European clinical guidance for the management of adrenal and extra-adrenal paraganglioma in children and adolescents: a consensus by the EXPeRT group	The European Cooperative Study Group for paediatric rare tumours	Paediatric PPGLs (9)
2026	Japan Endocrine Society clinical practice guideline for the diagnosis and management of pheochromocytoma and paraganglioma 2025	Japan Endocrine Society	Broad diagnosis, genetics, and treatment (52)

**Figure 1:**
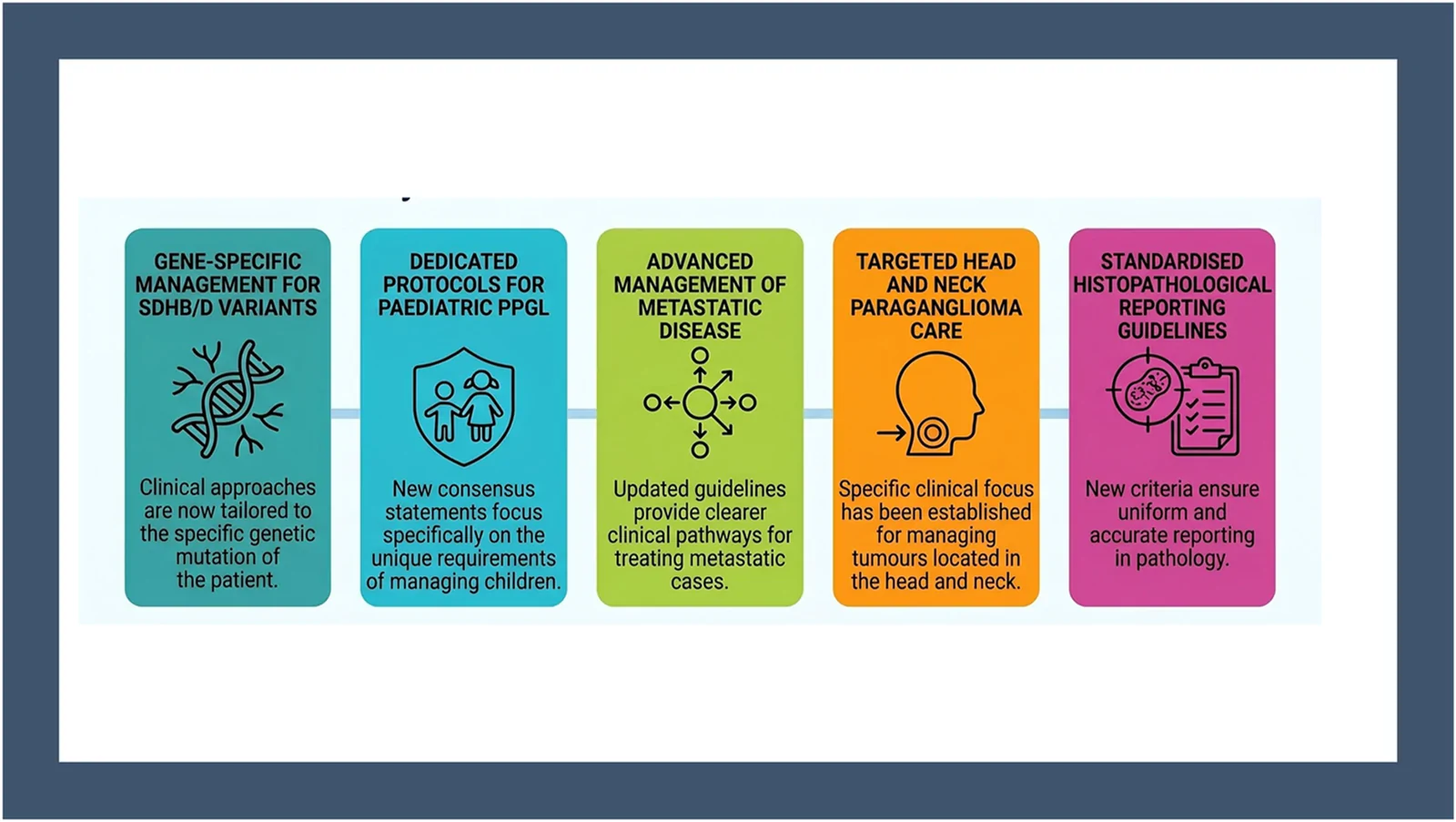
Key clinical guidance changes in the past 5–10 years reviewed in this article.

### Genotype-tailored management of *SDHB* and *SDHD* pathogenic variant carriers

The management of PPGLs has shifted towards a genotype-first approach, particularly for patients with germline pathogenic variants in *SDHB* and *SDHD*. These variants carry distinct clinical profiles: *SDHB* is associated with a high risk of metastatic disease (30–40%) and predominantly sympathetic extra-adrenal paragangliomas, while *SDHD* variants typically manifest as multifocal, slow-growing head and neck paragangliomas (HNPGLs) with a lower metastatic risk (approximately 5%) (15, 16).

#### Initial assessment

The management of patients with PPGLs who carry *SDHB* or *SDHD* pathogenic variants is complex and requires a personalized approach guided by an interdisciplinary team of experts.

Initial screening protocols are similar in patients harbouring a tumour (6, 7) and for individuals identified as asymptomatic carriers through family screening (5). The primary goal for asymptomatic carriers is early detection to improve long-term outcomes. Screening should begin earlier for *SDHB* mutation carriers (between 6 and 10 years of age) due to the risk of aggressive childhood-onset tumours. For *SDHD* (paternal transmission), *SDHA*, and *SDHC* carriers, the first screening is recommended between 10 and 15 years of age.

In patients with a paraganglioma and an *SDHx* mutation, initial work-up should help the clinician to answer the following questions: does the patient have i) a secretory tumour, ii) multiple synchronous paraganglioma tumours, iii) distant metastases related to the paraganglioma, and iv) any other *SDHx-*related tumours (e.g. gastrointestinal stromal tumour or GIST and clear cell renal cell carcinoma (ccRCC)). Based on this assessment, a multidisciplinary team will be able to determine the best treatment option.

The initial work-up should include a clinical review with a thorough history of symptoms of catecholamine secretion (headaches, sweating, palpitations, and weight loss), ambulatory blood pressure measurement alongside a formal evaluation of the cranial nerves. Biochemical testing involves measuring plasma or urinary metanephrines (metanephrine and normetanephrine), with a preference for plasma-free metanephrines over urinary tests due to higher diagnostic accuracy (17). Measurement of plasma 3-methoxytyramine (the metabolite of dopamine) is also recommended, particularly for *SDHB* and *SDHD* head and neck paragangliomas (HNPGLs), which may only show elevations in this specific metabolite (12, 18). Imaging must encompass a whole-body scan from the skull base to the pelvic floor. Regarding anatomical imaging, MRI is the first-line choice for paediatric patients and asymptomatic carriers to minimize cumulative radiation exposure, while CT is less costly and reserved for perioperative planning. For *SDHD* HNPGLs, head and neck MRI with angiography (MRA) is the most sensitive technique for staging and screening for multifocality. Functional imaging is recommended at first initial evaluation for patients with and without diagnosed tumours (5, 6, 7). ^68^Ga-radiolabelled somatostatin analogues PET-CT (^68^Ga-SSTR PET-CT) is the gold standard for initial staging. It has a sensitivity approaching 100% for HNPGLs and is superior for detecting metastatic disease in patients with *SDHB* pathogenic variants (19). As alternatives, ^18^F-FDG PET-CT is sensitive for detecting metastatic disease (20), and ^18^F-FDOPA PET-CT is also highly accurate for *SDHD* HNPGLs but may miss sympathetic tumours outside the head and neck (21, 22). ^131^I-MIBG scintigraphy lacks sensitivity and should not be used as a first-line diagnostic tool for *SDHx* carriers (16, 23).

#### Surgical management

Across both genotypes, all major management decisions should be carried out by an expert, interdisciplinary team. This team evaluates the risk–benefit balance, considering the tumour’s functional status, the likelihood of achieving a margin-negative surgical resection, and the potential for treatment-related morbidity.

Surgery with curative intent is the first-line treatment for localized disease, but the approach varies significantly by genotype and localization. In all patients with an *SDHx* mutation, if multifocal disease is present, functional (catecholamine-secreting) retroperitoneal PPGLs are resected as a priority to prevent hypertensive crises. In patients carrying an *SDHB* mutation, due to the high risk of recurrence and metastasis, total resection is recommended. For large (>6 cm) or potentially invasive thoraco-abdominal tumours, an open approach is preferred over laparoscopy to allow for complete vascular/lymphatic dissection and to avoid capsular disruption. Cortex-sparing adrenal surgery is generally not recommended for *SDHB*-related phaeochromocytomas because of the low risk of bilateral disease and the higher risk of metastatic spread. For patients with an *SDHD* mutation, the most likely phenotype is head and neck paragangliomas, which may be multifocal, with the ‘first, do no harm’ axiom that will be discussed in part III.

#### Follow-up

Lifelong surveillance is mandatory for both *SDHB* and *SDHD* mutation carriers due to the persistent risk of new tumours or late-onset metastasis. Following surgery with curative intent, 24-h urinary metanephrines should be checked 2–8 weeks postoperatively for functional tumours. Initial follow-up imaging (CT or MRI) is typically performed within 3–6 months of surgery. For patients who undergo curative surgery and have normal imaging at 3–6 months post-surgery, further surveillance can be carried out at intervals of 6–12 months initially and extended to longer intervals if the patient remains asymptomatic and tumour-free. [^68^Ga]-SSTR PET-CT is recommended in patients with an *SDHB* mutation at 6 months, especially if not performed preoperatively and can be considered during follow-up of patients with an *SDHD* mutation on an individual basis every 2–3 years (6, 7).

For asymptomatic carriers of pathogenic *SDHB* and *SDHD* variants, clinical assessment and metanephrine measurements should be performed annually (5). Imaging relies predominantly on non-ionizing whole-body MRI and is recommended every 2–3 years. PET-CT can be used as an alternative to MRI every 5–6 years depending on local practice and the clinician’s preference. Finally, in mutation carriers who have never developed a tumour and remain asymptomatic, follow-up intervals may be increased after age 70, and surveillance may potentially stop at age 80 (5) (Fig. 2).

**Figure 2:**
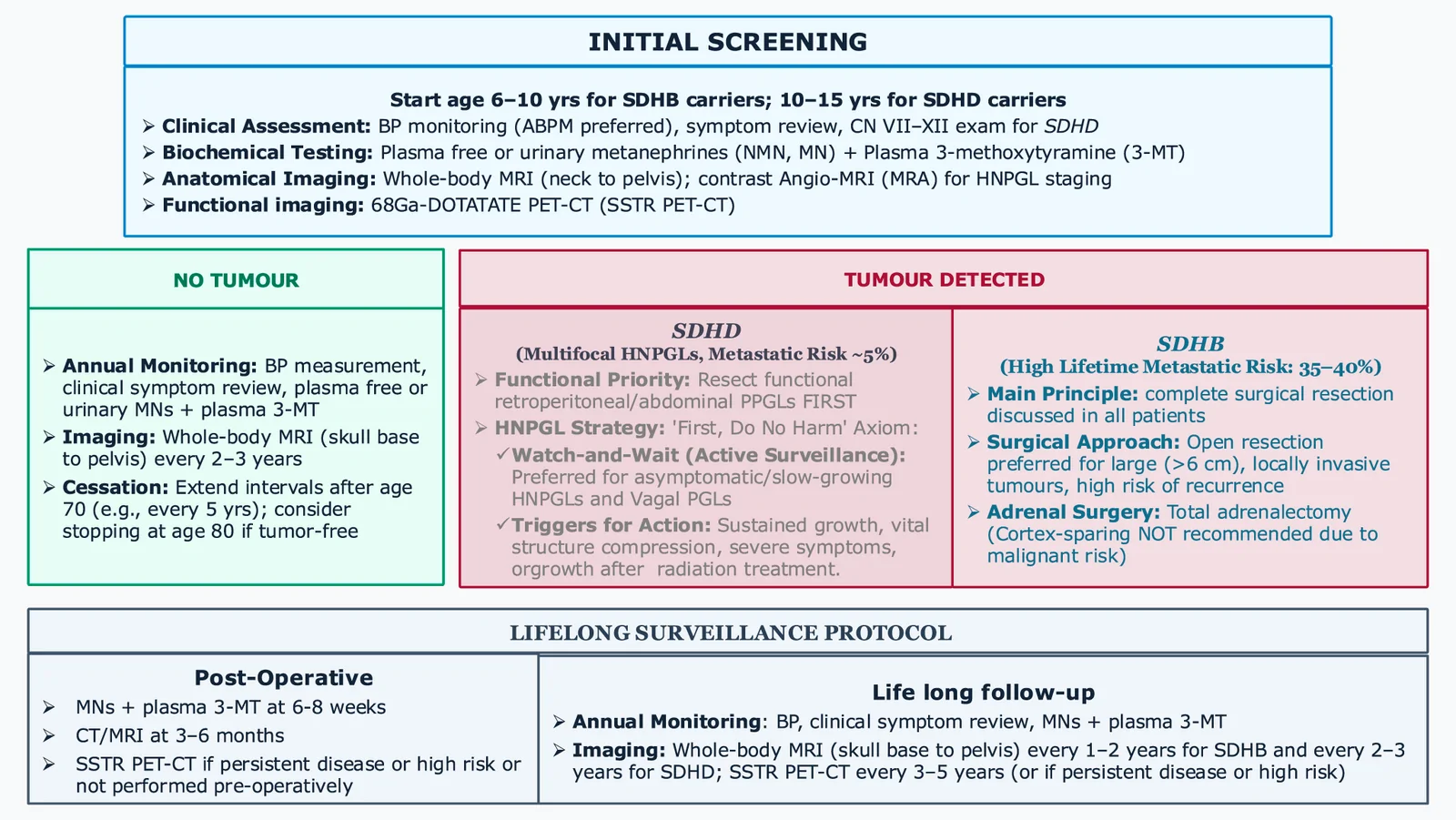
Genotype-led management algorithm for patients with a pathogenic variant in *SDHB* and *SDHD* GENES. MN(s), metanephrines; NMN, normetanephrine; CN, cranial nerve.

### Specific approach for children versus adults

Paediatric PPGLs are rare with an annual incidence of 0.5–2 cases per million children. Paediatric PPGLs present unique clinical challenges compared with adults, requiring specialized multidisciplinary teams including paediatric oncologists, endocrinologists, and geneticists. These key diagnostic and management challenges have been tackled in two recent international guidance statements.

Up to 80% of paediatric PPGLs are hereditary compared with only 35–50% in adults; therefore, universal germline genetic testing is strongly recommended for all children regardless of family history (8, 9, 24, 25). While adults can present with either paroxysmal or sustained hypertension (26), children typically present with sustained hypertension due to continuous catecholamine excess. Children may also present with less typical symptoms such as behavioural symptoms including social withdrawal, hyperactivity, or attention deficit. Finally, as children may have smaller tumours compared with adults, given what is known about the average doubling times of PPGLs, the concentration of catecholamines may still be too low to cause the typical symptoms of catecholamine excess, and this may lead to a delayed diagnosis in some children (27, 28, 29).

Diagnosis in both adult and paediatric groups relies on liquid chromatography assays for metanephrines, but plasma testing is preferred in children to avoid the difficulty of 24-h urine collections (9, 17, 30). Crucially, laboratories must apply age-specific reference intervals for paediatric plasma metanephrines because baseline concentrations naturally vary throughout childhood development (31). For anatomical localization, MRI is often favoured over CT as the initial modality in children to minimize lifetime exposure to ionizing radiation as discussed earlier. Preoperative alpha-adrenoceptor blockade must be initiated earlier in children than in adults and at least 14 days before surgery (9). Complete surgical resection is the primary curative treatment, but minimally invasive cortical-sparing partial adrenalectomy is highly prioritized in children to prevent lifelong adrenal insufficiency, especially given their higher rate of bilateral tumours. However, this cortical-sparing approach is generally also avoided in paediatric patients with *SDHB* mutations due to their higher risk of malignant transformation and local recurrence (8, 9). Finally, because paediatric patients are more likely than adults to develop metachronous metastatic tumours or multiple primary lesions over time, they require lifelong, genotype-specific clinical and radiological surveillance (8, 9).

### Head and neck PGL guidelines in patients with pathogenic SDHD variants

For patients with *SDHD*-related head and neck paragangliomas (HNPGLs), the ‘watch-and-wait’ strategy – also referred to as active surveillance or watchful waiting – is a cornerstone of management based on the axiom ‘first, do no harm’. Because *SDHD*-related tumours are typically slow-growing and benign (32, 33), this approach aims to balance tumour control against the high risk of treatment-induced morbidity, such as permanent cranial nerve damage.

Like in most sporadic HNPGLs, an initial period of observation is often appropriate to characterize the individual behaviour of a tumour. Ideal candidates for observation are newly diagnosed patients with jugular, vagal, or carotid paragangliomas who do not have urgent indications for intervention (such as brainstem compression, severe pain, or bleeding). Median growth rates for these lesions can be as low as 1 mm per year, and studies have shown that approximately 60% of *SDHD*-related paragangliomas do not progress during surveillance (33). Observation is specifically recommended for vagal tumours because surgical resection almost always results in vagal nerve sacrifice and vocal cord paralysis. In patients with bilateral tumours, the aim is to prevent the devastating outcome of bilateral cranial nerve palsies, which can lead to the need for a tracheostomy. The goal of surveillance is to document the disease course and detect changes that might necessitate a shift to active treatment. Treatment-naive patients should undergo imaging at 3–6 months and again at 1 year after the initial diagnosis to establish the growth pace (7). Then, if the disease remains stable (for >12 months), annual assessment is typically sufficient. Observation is discontinued in favour of intervention (surgery or radiotherapy) if certain triggers are met, such as sustained or rapid growth of the tumour, compression of vital structures in the head and neck, development of new or progressive cranial neuropathies, and significant mass effects or pain.

The cranial nerve status is important in the decision-making process. For vagal PGL, surgery is generally deferred until the vocal cord is already immobile to avoid unnecessary nerve sacrifice. In cases of multifocal disease, surgery is planned to avoid devastating bilateral cranial nerve palsies; if a patient already has a nerve deficit on one side, intervention on the contralateral side is handled with extreme caution.

Finally, surgical resection is indicated in cases of progression after radiation therapy. On the other hand, radiotherapy or radiosurgery can be considered in cases of residual tumour growth or incomplete surgery.

### Histopathological classification of PPGLs

Since the 2017 World Health Organization (WHO) classification, the terms ‘benign’ and ‘malignant’ have been abandoned for PPGLs. The current 2022 WHO classification advises that all PPGLs should be considered malignant neoplasms with variable metastatic potential (34, 35). The challenge in clinical practice is to predict the metastatic potential for the individual patient. To assist with this prediction, several independent pathological grading and scoring systems have been developed. PASS (Pheochromocytoma of the Adrenal gland Scaled Score), introduced in 2002, is based entirely on histological criteria and was originally designed solely for adrenal tumours (PCC). The strength of the PASS score is a high negative predictive value for the absence of metastatic behaviour; however, its clinical utility is limited by considerable interobserver variation when applying the histological criteria (36). The GAPP (Grading system for Adrenal Pheochromocytoma and Paraganglioma) score is a grading system which calculates a maximum score of 10 by evaluating four histological criteria (growth pattern, cellularity, necrosis, and vascular or capsular invasion), combined with the Ki67 labelling index and the tumour’s catecholamine secretion type. The GAPP score classifies tumours into well differentiated (score 0–2), moderately differentiated (score 3–6), and poorly differentiated (score 7–10) categories, with a higher score denoting a less well-differentiated tumour and an anticipated higher risk of recurrent disease and distant metastasis. A modified version of GAPP has also been proposed that incorporates succinate dehydrogenase subunit B (SDHB) immunohistochemistry (37). Finally, the COPPS (COmposite Pheochromocytoma/paraganglioma Prognostic Score) is a more recently proposed system that integrates clinicopathological criteria with immunohistochemistry. It evaluates tumour size (>7 cm), necrosis, and vascular invasion, alongside the loss of S100 and/or SDHB immunohistochemical staining. A COPPS of 3 or higher is correlated with reduced progression-free survival and was found to predict metastasis with 100% sensitivity and 95% specificity in initial studies (38).

The 2022 WHO classification does not officially endorse any of these specific grading systems, noting that while they have benefits, higher scores can sometimes lead to an overestimation of metastatic risk. However, the WHO does not discourage pathologists from using these scores, but suggests they are most optimally utilized when combined with molecular risk factors, such as *SDHB* or *ATRX* pathogenic variants, which are significant indicators of adverse biology and metastatic behaviour. The recent WHO update also promotes immunohistochemistry for accurate diagnosis and genetic screening, including the routine use of SDHB immunohistochemistry as a critical surrogate biomarker to identify patients with underlying mutations in one of the *SDHx* genes, which are strongly linked to metastatic behaviour (34, 39). A recent protocol was published outlining the optimal practices for handling and reporting of PPGL specimens and pays tribute to the rate of discovery in this field and the need for uniform histopathological and molecular reporting that can provide clear and uniform information to pathologists and clinicians (14).

The 2022 WHO classification also introduced several significant updates to the PPGL terminology. The main changes are listed as follows: i) reclassification of ‘paraganglioma-like tumours’, including cauda equina paraganglioma, which has been renamed to cauda equina neuroendocrine tumour, and gangliocytic paraganglioma, which has been renamed to composite gangliocytoma/neuroma and neuroendocrine tumour (CoGNET); ii) renaming of precursor lesions: previously, nodular lesions in the adrenal medulla smaller than 1 cm (frequently seen in patients with MEN2 syndrome) were termed ‘nodular hyperplasia’, but as studies have shown that these small nodules share the same molecular markers as fully developed tumours, the 2022 WHO guidelines formally replaced the term ‘nodular hyperplasia’ with ‘micro-phaeochromocytoma’; and iii) renaming phaeochromocytoma: to unify the conceptual framework of these tumours while acknowledging the clinical importance of anatomic site, the WHO now formally defines a phaeochromocytoma as an ‘intra-adrenal paraganglioma’ (34, 35).

### Managing metastatic PPGLs

#### Current standard of care

The management of patients with metastatic PPGLs with an *SDHB* mutation is complex and requires a multidisciplinary team approach, ideally at high-volume medical centres. *SDHB* pathogenic variants are associated with the highest rates of aggressive behaviour, with 30–40% of patients developing metastatic disease (40, 41). Other factors are independently associated with a higher risk of developing metastases, like a primary tumour size ≥5 cm, a primary tumour located in the abdominopelvic area (extra-adrenal sympathetic PGL), a predominant secretion of noradrenaline and a high plasma level of 3-methoxytyramine, and an early age of onset, specifically before 20 years old (42).

Treatment selection depends on the rate of disease progression and the tumour burden.

For asymptomatic patients with a low tumour burden, active surveillance may be appropriate in select individuals. All patients with catecholamine-secreting tumours must be managed with alpha-adrenoceptor blockers to prevent cardiovascular complications, especially during invasive or systemic treatments which may trigger a catecholamine crisis (43, 44). Surgery of the primary tumour may be recommended even in the presence of synchronous metastases, as it may offer symptomatic benefit from local compressive effects of the primary tumour and may afford better control of catecholamine excess in the setting of a secretory tumour (45, 46). External beam radiation could be an effective palliative treatment for symptomatic bone metastases. Locally ablative therapies including radiofrequency ablation and radioembolization can be considered for select patients, typically those with oligometastatic disease confined to bone or liver.

In patients with progressive disease, systemic therapies are typically recommended, and the selection of systemic therapy is influenced by disease burden, rate of disease progression, patient symptoms, and increasingly by the underlying genetic driver (both germline and somatic drivers) of the PPGL.

Targeted radionuclide therapy is preferred for patients with slow-to-moderate progression and moderate-to-high tumour burden (6). The use of ^131^I-MIBG is hampered by its limited availability and low or heterogeneous uptake in patients with pathogenic *SDHB* variants. ^177^Lu-DOTATATE is more widely used due to its availability and the high somatostatin receptor expression in PPGLs, including *SDHB*-mutated tumours. In a phase II study by Lin *et al*. (47), the efficacy and safety of ^177^Lu-DOTATATE was prospectively evaluated in 36 patients with progressive metastatic PPGLs within 12 months, divided into two cohorts (*SDHx*-mutated or apparently sporadic). The treatment was well tolerated, with a partial response in 13.8% of patients at 8 weeks and a best response achieved on average 11.0 months after completing ^177^Lu-DOTATATE. Nevertheless, patients with *SDHx* pathogenic variants appeared to have significantly lower response rate and progression-free survival than sporadic patients (12.9 months for *SDHx* vs 24.3 months for sporadic) (47). This result will need to be confirmed in larger prospective studies focused on stratifying results based on initial tumour burden and rate of disease progression.

Chemotherapy with CVD (cyclophosphamide, vincristine, and dacarbazine) is reserved for rapidly progressive disease or for those patients with high visceral tumour burden. Temozolomide, a dacarbazine oral counterpart, is an alternative second-line therapy, with some evidence suggesting superior responses in patients with underlying *SDHB* mutations (48).

#### Emerging therapies

In the FIRSTMAPPP study (the first randomized controlled trial conducted for patients with progressive metastatic PPGLs), 78 adults with sporadic or inherited progressive metastatic PPGLs were enrolled across 14 European centres and randomly assigned to receive either 37.5 mg of sunitinib per day or a placebo (49). Sunitinib significantly extended the median progression-free survival to 8.9 months vs 3.6 months in the placebo group, with a 36% progression-free survival (PFS) rate at 12 months, compared with 19% in the placebo group. Moreover, a higher response rate was observed in patients harbouring a pathogenic germline *SDHB* variant.

Belzutifan, a small-molecule HIF-2α inhibitor, is emerging as a hopeful targeted therapy for patients with metastatic PPGLs. In the LITESPARK-015 phase 2 international single-arm trial (50), 72 patients with advanced or metastatic disease received 120 mg per day of belzutifan, providing an objective response rate (ORR) of 26% (all partial responses), and the disease control rate reached 85%. The median PFS was 22.3 months. Similar to sunitinib, patients with an *SDHB*-mutated tumour seemed to have a higher response rate (38 vs 25%). The tolerance seems to be higher for belzutifan than for sunitinib (only 3% of treatment discontinuation for adverse effects versus 14% under sunitinib). Nevertheless, the belzutifan study was not randomized, and progression before treatment was not an inclusion criterion.

In summary, for patients with progressive metastatic disease, in combination with locoregional therapies, when possible, targeted radionuclide therapy using ^177^Lu-DOTATATE or ^131^I-MIBG is a suitable first-line option for patients with slow-to-moderate progression, while sunitinib and belzutifan could be considered for more aggressive tumours or as second-line therapy, especially in patients with underlying pathogenic *SDHB* variants. The sequence of therapies may change in coming years as more evidence becomes available to support the benefit and preferred sequence of emerging therapies. Finally, chemotherapy is reserved for patients with very high disease volume or rapidly progressive disease or for those patients who have progressed on earlier lines of treatment, but who remain clinically fit enough to be considered for systemic chemotherapy.

### Long-term follow-up for sporadic PPGL cases

Guidelines for monitoring recurrence in patients with PPGLs emphasize that all such tumours have malignant potential and require long-term surveillance (1). The follow-up strategies are tailored based on the tumour’s location, genetic background, and specific risk factors. It is generally accepted that patients with hereditary PPGLs will require lifelong follow-up to monitor for disease recurrence and the development of new synchronous primary tumours or conditions related to their genetic diagnosis. The duration and frequency of follow-up for patients with sporadic PPGLs is less well defined; however, recent data suggest a delayed risk of recurrence indicating that prolonged follow-up should be considered for patients with sporadic PPGLs and lifelong follow-up may be necessary for some individuals with higher clinical risk factors.

#### Sporadic head and neck paragangliomas

The prevalence of recurrent disease in patients with head and neck paragangliomas (HNPGLs) is almost three times higher than in other PPGLs (51). However, unlike hereditary forms of the disease, recurrences in sporadic HNPGL (spHNPGL) cases are typically limited to the head and neck regions, and these patients do not develop sympathetic tumours in other parts of the body. Over 75% of recurrences in sporadic HNPGLs are diagnosed within 15 years of the initial tumour diagnosis, while metastasis occurs in approximately 9% of spHNPGL cases and the prevalence of metastatic disease is similar between patients with spHNPGLs and those with hereditary HNPGLs (7, 51). The carotid body location and male sex were identified as potential predictors of recurrence on univariate analysis for patients with spHNPGLs; however, a positive *SDHx* gene mutation status was the only predictor of recurrent disease on multivariate analysis in one large study (12). Due to the specific clinical behaviour of spHNPGLs, management and surveillance strategies are tailored differently from other types of paragangliomas. As most recurrences in patients with spHNPGLs happen within the first 15 years, a minimum follow-up period of at least 15 years is strongly recommended (12). Lifelong annual follow-up can be considered for some patients with spHNPGLs, including young patients without a genetic diagnosis, patients with very large tumours, or those with carotid body tumours. Because recurrences are confined to the head and neck, routine post-treatment surveillance can be simplified for patients with spNHPGL. Anatomical imaging, such as a contrast-enhanced MRI or CT, should focus solely on the head and neck region. Whole-body functional imaging (like PET/CT) is not routinely required unless there is suspicion of metastasis, or if the tumour is in the carotid body. Follow-up intervals for sporadic cases can be individualized based on local signs, symptoms, and the functionality of the tumour. For biochemically inactive tumours, imaging should be considered within 6 months of initial surgery or radiotherapy treatment and then every 1–3 years using contrast-enhanced head and neck MRI. For patients with functional tumours (this includes patients with elevated 3-methoxytyramine or plasma or urine normetanephrine levels), biochemistry should be repeated at similar intervals to imaging unless there are new signs or symptoms (Table 3).

**Table 3:** Long-term surveillance strategies for sporadic PPGLs.

Tumour type	Duration of surveillance	Monitoring strategy & intervals
Sporadic HNPGLs	At least 15 years	Flexible intervals (1–3 yearly) Focused imaging (MRI/CT) of the head and neck only Biochemistry: pMETS/UMETS ± plasma 3MT only if elevated prior to surgery
Sporadic PPGLs (excluding HNPGLs)	At least 10–15 years Lifelong if high-risk*	Biochemically active: pMETS/UMETS ± plasma 3MT every 6–12 months Biochemically inactive: Imaging (CT/MRI) every 1–2 years

PMETS, plasma-free metanephrines; UMETS, 24-h urine metanephrine and normetanephrine; plasma 3MT, plasma 3-methoxytyramine.

* High-risk features for sporadic PPGLs include young age, primary tumour ≥ 5 cm, extra-adrenal location, or a noradrenergic/dopaminergic phenotype.

#### Sporadic PPGLs

The overall prevalence of recurrence in patients with sporadic phaeochromocytomas and paragangliomas (PPGLs) is approximately 14.7% and, looking specifically at sporadic PCC, the recurrence rate is only slightly lower at 14.2% (52). Disease recurrence (local and metastatic) for patients with sporadic PPGLs can occur after a prolonged disease-free period; roughly 65.8% are diagnosed within the first 5 years, but 29.1% are diagnosed at least 10 years after the initial tumour diagnosis, and 17.7% are diagnosed 15 years or more after the first diagnosis (53). The risk of recurrence is heavily influenced by specific clinical and biochemical features. Multivariable analyses have demonstrated that a noradrenergic/dopaminergic biochemical phenotype, a primary tumour diameter of >5 cm, and an extra-adrenal location are independent predictors of recurrent disease in sporadic cases. Younger age at initial diagnosis may also be a risk factor. Patients with small (<3 cm) sporadic adrenergic tumours have a notably low recurrence rate of just 3.2% (53).

Because recurrences can occur decades after the initial surgery, guidelines mandate extended follow-up tailored to the patient’s risk profile. Surveillance is recommended for at least 10 years in all patients operated on for a sporadic PPGL (13, 52). However, because nearly 18% of recurrences happen beyond 15 years, extending follow-up past 15 years may be justified (53). For sporadic cases deemed high-risk (e.g. young age, large tumours ≥5 cm, extra-adrenal location, or noradrenaline dominance), lifelong annual follow-up is strongly recommended (53). The nature and intensity of the follow-up should be individualized based on the primary tumour’s characteristics. Patients with high-risk features require strict monitoring, whereas surveillance might not need to be as exhaustive for those with low-risk profiles, such as small adrenergic tumours. For functionally active tumours, annual measurement of plasma or urinary fractionated metanephrines (and optionally 3-methoxytyramine) is the standard method to screen for local or metastatic recurrences and new tumours and should be repeated 3 months after surgery and then at 6–12-month intervals depending on the patient’s clinical risk profile.

If the primary sporadic tumour did not secrete catecholamines, biochemical tests are inadequate for surveillance. These patients should undergo imaging tests (such as CT or MRI) every 1–2 years. In addition, an initial imaging test should be performed 3–6 months postoperatively (13, 52, 53) (Table 3).

### Predicting metastatic PPGL risk: existing and emerging diagnostic tools

#### Clinical risk factors and histopathological and molecular markers

Recent advances in predicting the metastatic potential of PPGLs highlight a paradigm shift towards integrating traditionally accepted clinical risk factors and histopathological features with multi-omic profiling, liquid biopsies, and artificial intelligence. As all PPGLs possess metastatic potential, identifying high-risk patients preoperatively and at initial diagnosis is a clinical priority to facilitate stratification of long-term follow-up.

Clinical risk factors for metastasis include extra-adrenal localization, large tumour size (particularly >5 cm), locally invasive tumours, and tumours arising in individuals with pathogenic germline *SDHx* variants, particularly in the *SDHB* gene. Histopathological features indicating a higher malignant risk include vascular or capsular invasion, high mitotic rate (>3/10 HPF), nuclear pleomorphism, and confluent necrosis. In a recent prospective study on tumour samples and liquid biopsies from 231 consecutive patients with PPGLs, the immunohistochemical loss of succinate dehydrogenase subunit B (SDHB) was a more powerful predictor of an *SDHx* status, while the prediction of metastasis and lower metastasis-free survival was achieved by a combination of 4 biomarker tools, namely telomerase maintenance, detection of circulating miR-483-5p, plasma succinate level, and SDHB immunohistochemistry (54). Overexpression of the mini-chromosome maintenance 6 (MCM6) protein, which is involved in DNA replication, has been associated with a higher incidence of distant organ metastasis and shows promise as a novel prognostic marker, though larger cohort studies are needed. In addition, tumours harbouring MAML3 fusion anomalies (detectable via nuclear overexpression) exhibit a distinctive, highly vascular tumour microenvironment and are linked to aggressive clinical behaviour (55, 56).

While canonical *TERT* promoter point mutations are known drivers of metastasis, they are relatively rare in PPGLs. Recent whole-genome sequencing has revealed that somatic structural rearrangements (such as intra-chromosomal deletions and translocations) can modulate enhancers close to the *TERT* promoter, leading to abnormal activation of *TERT* in metastatic PPGLs. Hypermethylation of the *TERT* promoter is a highly accurate prognostic biomarker of metastatic PPGLs (39, 57). Somatic pathogenic mutations in the *ATRX* gene are also significantly associated with metastatic progression and telomere dysregulation. Tumours with *ATRX* alterations consistently exhibit the alternative lengthening of telomeres (ALT) phenotype, characterized by the presence of C-circles and longer telomeres (39). In metastatic patients treated with DNA alkylating chemotherapies (such as dacarbazine or temozolomide), multi-omic profiling has identified acquired resistance mechanisms, including MGMT overexpression and mismatch repair (MMR) deficiency leading to hypermutation (e.g. via *MLH1* or *MSH6* mutations) (39).

#### Metabolomic and liquid biopsy biomarkers

Elevated plasma succinate (>4.94 μM) correlates with tumour burden and is highly effective at identifying patients carrying underlying *SDHx* mutations (which confer a high metastatic risk). It provides a rapid, non-invasive screening tool with 65% sensitivity and 92% specificity (54). This test is relatively inexpensive and offers a potentially affordable method to rapidly identify underlying pathogenic *SDHx* variants for individuals diagnosed in countries where genetic testing is not offered as current standard of care.

Liquid biopsies have identified miR-483-5p as the single best circulating microRNA classifier for metastatic status. Elevated levels of miR-483-5p correlate with tumour burden and predict the risk of developing future metastases (54). Finally, elevated plasma methoxytyramine indicates an undifferentiated, high-risk tumour phenotype with high specificity (85%); however, if interpreted in isolation, the sensitivity of plasma methoxytyramine is limited (52%) and as a biomarker for metastatic potential, it is best utilized alongside other clinical features (42).

#### Machine learning (ML) prediction models

A significant advance in predictive accuracy has come from machine learning models that integrate diverse datasets and can complement or augment the judgement of human clinical specialists. A model utilizing nine routine features – plasma methoxytyramine, metanephrine, normetanephrine, age, sex, previous PPGL history, tumour location, tumour size, and multifocal disease – achieved a highly accurate prediction of metastasis (AUC of 0.942). Notably, this model achieved excellent diagnostic performance without requiring genetic data, making it highly practical for routine clinical use (42). Another multivariate model combined more advanced biological variables (circulating miR-483-5p, *TERT* promoter methylation, SDHB IHC, and plasma succinate) with standard clinical data (tumour size, age, site, germline status) and demonstrated diagnostic accuracy (AUC-ROC of 0.97) for predicting metastatic disease (54).

## Summary

This document provides an in-depth review of recent clinical advancements and international consensus guidelines regarding the diagnosis, treatment, and long-term management of PPGLs. The management of these rare neuroendocrine tumours is increasingly driven by specialized, multidisciplinary care. International collaborations fostered over the past decade have acted as the major catalyst for significant research advances and expert consensus guidelines in this field. Key findings from this article include the following: i) modern care of PPGLs is heavily driven by ‘genotype-first’ clinical management; ii) the importance of agreed specialized paediatric diagnostic and management protocols, acknowledging key differences in the biology of paediatric versus adult PPGLs; iii) systemic therapy is increasingly tailored based on the patient’s underlying genetic driver; and iv) the prediction of metastatic risk is evolving and improving with the integration of novel molecular biomarkers and machine learning models.

## Declaration of interest

CLL and RTC have no conflict of interest for this article.

## Funding

RTC was supported by the Cambridge NIHR Biomedical Research Centre (NIHR203312). The views expressed are those of the authors and not necessarily those of the NIHR or the Department of Health and Social Care.
